# Contribution to the treatment of nausea and emesis induced by chemotherapy in children and adolescents with osteosarcoma

**DOI:** 10.1590/S1516-31802006000200003

**Published:** 2006-03-02

**Authors:** Flavio Augusto Vercillo Luisi, Antônio Sérgio Petrilli, Cristiana Tanaka, Eliana Maria Monteiro Caran

**Keywords:** Vomiting, Nausea, Child, Adolescent, Drug therapy, Vômito, Náusea, Criança, Adolescente, Quimioterapia

## Abstract

**CONTEXT AND OBJECTIVE:**

Chemotherapy-induced emesis is a limiting factor in treating children with malignancies. Intensive chemotherapy regimens along with emetogenic drug administration have increased the frequency and severity of emesis and nausea. Our study was designed to consider the importance of this problem and the need for improvement in emesis treatment for patients receiving chemotherapy. Our objective was to compare the efficacy and safety of the antiemetic drug granisetron and a regimen of metoclopramide plus dimenhydrinate.

**DESIGN AND SETTING:**

Open, prospective and randomized study at Instituto de Oncologia Pediátrica, Department of Pediatrics, Universidade Federal de São Paulo.

**METHODS:**

From February to August 1994, 26 patients (mean age: 14 years) with osteosarcoma received 80 chemotherapy cycles of iphosphamide (2,500 mg/m^2^) plus epirubicin (75 mg/m^2^) or carboplatin (600 mg/m^2^), or epirubicin (75 mg/m^2^) plus carboplatin (600 mg/m^2^). Eighty chemotherapy treatments were analyzed regarding nausea and vomiting control. Patients were randomized to receive either a single dose of granisetron (50 µg/kg) or metoclopramide (2 mg/kg) plus dimenhydrinate (5 mg/kg infused over eight hours). Emesis and nausea were monitored for 24 hours by means of the modified Morrow Assessment of Nausea and Emesis. Statistical analysis utilized the chi- squared, Student t and Mann-Whitney tests, plus data exploration techniques.

**RESULTS:**

62.5% of the patients undergoing chemotherapy responded completely to granisetron, whereas 10% responded to metoclopramide plus dimenhydrinate (p < 0.0001). No severe adverse reactions were found in either of the treatments given.

**CONCLUSION:**

In children and adolescents with osteosarcoma, granisetron was safe and more efficient than metoclopramide plus dimenhydrinate for controlling chemotherapy-induced emesis and nausea.

## RESUMO

**CONTEXTO E OBJETIVO:**

A êmese induzida por quimioterapia é fator limitante no tratamento de crianças com câncer. O uso de quimioterapia com drogas emetogênicas tem aumentado a freqüência desse efeito colateral. O objetivo é comparar a eficácia e a toxicidade do granisetron às da combinação de altas doses de metoclopramida e dimenidrato em crianças com osteossarcoma utilizando a mesma quimioterapia.

**TIPO DE ESTUDO E LOCAL:**

Aberto, prospectivo, randomizado, realizado no Instituto de Oncologia Pediátrica, Departamento de Pediatria, Universidade Federal de São Paulo, Brasil.

**MÉTODOS:**

Entre fevereiro e agosto de 1994, 26 crianças com idade de 7 a 18 anos (média de 14 anos), recebendo quimioterapia para osteossarcoma, entraram no estudo. A quimioterapia consistiu de ciclos repetidos de: A) ifosfamida 2.500 mg/m^2^ + epirrubicina 75 mg/m^2^; B) ifosfamida 2.500 mg/ m^2^ + carboplatina 600 mg/m^2^; C) carboplatina 600 mg/m^2^ + epirrubicina 75 mg/m^2^. 80 tratamentos quimioterápicos foram avaliados para o controle de náuse e vômito. Os pacientes foram randomizados para receber dose única de granisetron (50 µ/kg) ou metoclopramida (2 mg/kg) mais dimenidrato (5 mg/kg) infundidos por oito horas. Êmese e náusea foram monitoradas por 24 horas por meio de escore de MANE (*Morrow Assessment of Nausea and Emesis*). Foram utilizados testes de Qui-quadrado, t e Mann Whitney, além da técnica de análise exploratória de dados.

**RESULTADOS:**

O granisetron induziu resposta completa em 62,5% dos pacientes submetidos aos tratamentos quimioterápicos comparado a apenas 10% obtidos com a combinação de metoclopramida associado ao dimenidrato (p < 0,0001).

**CONCLUSÕES:**

Concluímos que o granisetron é droga segura e eficiente em crianças com osteossarcoma superior à associação de metoclopramida e dimenidrato no controle de náuseas e vômitos induzidos por quimioterapia para osteossarcoma em crianças.

## INTRODUCTION

Because of the great progress in medical research over recent decades, chemotherapy has become remarkably important in the fight against cancer, especially for pediatric patients. However, its administration may cause a series of negative side effects such as myelosuppression, mucositis, hyperthermia, nausea and vomiting. Perhaps the most unpleasant ones are nausea and vomiting, which may last for a few days following chemotherapy and may sometimes be difficult to control. These adverse effects gain in importance because they frequently limit the use of chemotherapy and may cause some patients, particularly adolescents, to refuse to continue treatment.

The use of certain drugs such as cisplatin, dacarbazine, cyclophosphamide, doxorubicin, epirubicin, iphosphamide and carboplatin, along with polychemotherapy regimens, gives rise to a significant vomiting effect. This may have the following complications: dehydration, electrolyte disturbances,^1^ Mallory-Weiss syndrome and psychological depression, and these have a large impact on patients' quality of life.^2^ In order to prevent and treat nausea and vomiting, the use of antiemetics is recommended.

Conventional doses of metoclopramide do not control chemotherapy-induced nausea and vomiting efficiently. Nevertheless, Gralla et al.^3^ showed that high doses of endovenous metoclopramide (2 mg/kg) were effective in controlling cisplatin-induced emesis. Therefore, metoclopramide was considered to be the drug of choice for patients treated with cisplatin. However, this antiemetic drug is associated with some adverse effects, especially extrapyramidal reactions, which are most frequently found in children and adolescents.^4^ In 1984, Terrin et al.^5^ showed that diphenhydramine was effective in treating extrapyramidal reactions from other dopaminergic antagonists by reverting neurological reactions in children to whom metoclopramide was given, within a few minutes. In 1985, Allen et al.^6^ utilized an association of diphenhydramine and metoclopramide in order to control emesis during chemotherapy treatment in children with cancer, thereby decreasing its extrapyramidal effects.

Today, there are several antiemetics on the market. Some of them are similar to metoclopramide with fewer side effects. The development of drugs with high selectivity and strong affinity for 5-hydroxytryptamine- 3 (5HT3) receptors has opened new perspectives for antiemetic therapy.^7^ The agents that have been studied most are ondansetron, granisetron and tropisetron.^8,9^ Our experience with ondansetron has shown that it is effective and safe, with few side effects, but it has to be administered endovenously three times a day endovenously together with oral supplementation for an optimal result. Granisetron is safe and effective in controlling nausea and vomiting with no severe side effects. Its major advantage lies in the duration of its 24-hour effect, with no need for additional or supplementary oral doses.^10,11^ This characteristic is particularly important when treating children and adolescents who are more susceptible to toxicity.

Despite all the improvements in treating chemotherapy-induced nausea and vomiting, no standard pediatric antiemetics treatment has yet been established. We decided to conduct this study after considering the significant role that nausea and vomiting play in relation to this special group, as well as the need for assessing the effectiveness and side effects of antiemetics.

## OBJECTIVES

1. To compare the effectiveness and side effects of granisetron given as a single dose, versus doses of metoclopramide plus dimenhydrinate, for children and adolescents with osteosarcoma undergoing treatment using the same chemotherapy protocol;
2. To evaluate the modifications made to the Morrow Assessment of Nausea and Emesis (MANE) scale in order to adapt it for children undergoing outpatient treatment, for measuring the degree of the antiemetic effect.

## METHODS

This was an open, prospective and randomized study to assess the effectiveness and safety of two antiemetic regimens for children with osteosarcoma treated with chemotherapy. It was conducted within the Oncology section of the Department of Pediatrics, Universidade Federal de São Paulo — Escola Paulista de Medicina (Unifesp-EPM), São Paulo, Brazil, from February to August 1994. To be considered eligible, patients had to be under 20 years of age, with a diagnosis of metastatic or non-metastatic osteosarcoma based on anatomopathological examination, and they had to be undergoing chemotherapy treated in a day hospital. If patients presented with renal or hepatic abnormalities, or chronic vomiting, or were given oral antiemetics on the day chemotherapy was administered, they were excluded from the study. Informed consent for the patients to take part in the study was obtained from the persons responsible for them.

The patients were randomized to receive either 50 µg/kg of granisetron in a single dose administered over a five-minute period, or 2 mg/kg of metoclopramide plus an 8-hour infusion of 5 mg/kg of dimenhydrinate. Dimenhydrinate (diphenhydramine theoclate) was used as a substitute for diphenhydramine, which was not available. Drugs were administered via endovenous route, and neither the patients nor the members of their families knew which medication they were receiving. Emesis assessment was done on the basis of the following observations: anticipatory nausea and vomiting, nausea (presence or absence), dry heaving (number of episodes) and vomiting (frequency, duration and severity).

An objective assessment was made by either the investigating physicians or the nurses in the day hospital, and by the patients' parents or relatives when they were at home. The patients were reassessed in the hospital 24 hours after the beginning of the chemotherapy.

These assessments were based on the MANE scale (Morrow Assessment of Nausea and Emesis),^18^ which was modified and adapted by us for application to children. Originally, this scale was used to assess emesis in adults and consisted of a questionnaire comprising the following events: anticipatory nausea and vomiting, nausea, dry heaving, and vomiting after the beginning of chemotherapy. It considers quantifications of the duration of these events, by the adult patients, based on the length of time for which nausea and vomiting continue to be present. It is a six-point scale method for measuring symptoms from the beginning of chemotherapy treatment until 24 hours later.

The MANE scale had to be adapted in order to be applied to children, so that the presence of an outside observer of the children (a physician or nurse), and also their parents, could be included. The title "Modified MANE Scale" was chosen, considering that the scores given would have to give more attention to the assessments made by the observer and parents. It was left to the child to inform us whether nausea was present or absent at 2, 4, 6, 8, 12, 18 and 24 hours after the beginning of the chemotherapy treatment. We made these changes in order to homogenize the assessment and adapt the MANE scale to pediatric patients.

The following definitions were utilized in the assessment:

- Nausea was defined as a subjective sensation of repugnance in the upper gastrointestinal tract. It was usually a prodromal symptom of vomiting.
- Vomiting was a retrograde and vigorous expulsion of gastric contents.
- Dry heaving or gagging was a rhythmic involuntary breathing movement with no expulsion of gastric contents.
- Anticipatory vomiting is a conditioned response related to previous chemotherapy experience that occurs in nearly 25% of patients.^12^ It generally comes before chemotherapy administration and is resistant to the usual antiemetics.

Antiemetic efficacy was defined according to the following scale of points: 1 = nausea, 2 = dry heaving, 3 = vomiting. This assessment was made every two hours, and the final score was obtained by summing the points over the 24-hour period. The final score was classified into four different categories as follows:

- Complete response: 0
- Partial response: 1 to 10
- Minimal response: 11 to 20
- Absence of response: > 20

The chemotherapy regimens used in the study were established in accordance with the protocols for osteosarcoma treatment adopted by Escola Paulista de Medicina. The following drug and dose combinations were utilized:

- Epirubicin (75 mg/m^2^) plus iphosphamide (2,500 mg/m^2^)
- Epirubicin (75 mg/m^2^) plus carboplatin (600 mg/m^2^)
- Iphosphamide (2,500 mg/m^2^) plus carboplatin (600 mg/m^2^)

After receiving chemotherapy, the patients could either stay in the hospital (for socioeconomic reasons) or go back home. Twenty-four hours after beginning the treatment, those who had gone home returned to the hospital, and all the patients underwent a new assessment.

The statistical analysis performed was based on the chi-squared, Student t and Mann-Whitney tests, and also utilized data exploration techniques.

## RESULTS

### Assessment from the beginning of chemotherapy until the end of the eighth hour

Twenty-six patients were included in the study (15 males and 11 females), with a mean age of 14 years (range: 7-19). There was no statistically significant difference between the groups in relation to the mean age. Three patients were excluded from the study because they had been given oral antiemetics before the beginning of chemotherapy. Another two patients were excluded because they refused to receive high doses of metoclopramide. Other patients replaced these excluded patients, in accordance with the randomization procedure. A total of 80 chemotherapy treatments were given, and some of the children were part of the study more than once; some of them were considered in up to seven different events.

- Anticipatory nausea: In three chemotherapy treatments, the patients presented with nausea before administration. Two had been randomized to metoclopramide and the third to granisetron. There was no statistically significant difference between the groups in relation to this variable.
- Anticipatory vomiting: In five chemotherapy treatments, the patients presented with anticipatory vomiting. Three had been randomized to granisetron and two to metoclopramide. All of them had previously been exposed to chemotherapy.
- Nausea: Two hours after the beginning of chemotherapy, nausea was observed in 12.5% and 7.5% of the patients in the metoclopramide and granisetron groups, respectively. Four hours after the beginning of chemotherapy, a significant difference was observed between the groups. In the metoclopramide group, the incidence of nausea increased to reach 50% of the patients by the end of the eighth hour, whereas in the granisetron group it reached 7.5% of them. By the end of the study, the patients in the metoclopramide and granisetron groups presented with nausea in 32 (80%) and nine (22.5%) chemotherapy treatments, or at rates of 2.4 and 0.45 episodes/treat- ment, respectively. This result showed that the patients in the granisetron group had three times less nausea than did the patients in the metoclopramide group (p < 0.001).
- Dry heaving: The patients in the metoclopramide and granisetron groups presented with dry heaving in 22 (55%) and four (10%) chemotherapy treatments, or at rates of 1.3 and 0.125 episodes/treatment, respectively. This represents a mean of ten times fewer occurrences of dry heaving among patients in the granisetron group (p < 0.001).
- Emesis: The patients in the metoclopramide and granisetron groups presented with emesis in 33 (85%) and 13 (32.5%) chemotherapy treatments, or at rates of 2.175 and 0.500 episodes/treatment, respectively. After 4 hours of chemotherapy, these proportions reached 71.43% in the metoclopramide group, versus 33.3% in the granisetron group. Comparison of these two groups showed a statistically significant difference (p = 0.02).

### Assessment between 8 and 24 hours

It is important to assess what happened to patients during the period between the eighth and 24^th^ hours, because of the difference in how the drugs were administered. In the metoclopramide group, the infusion of the drug would finish upon reaching the end of the eighth hour, whereas in the granisetron group, the antiemetic was administered at time zero. In the granisetron group, it was expected that the effect of the drug would last for 24 hours, which would be different from the metoclopramide group, for which an additional dose six hours later is recommended.

- Nausea: In the granisetron group, patients still complained about nausea in only three chemotherapy treatments (0.075 episodes/treatment) at the end of the 12^th^ hour. In the metoclopramide group, patients kept complaining about nausea in 18 treatments (0.45 episodes/treatment). This was a statistically significant difference (p = 0.0001) signifying that patients in the metoclopramide group complained about nausea six times more frequently than did patients in the granisetron group in the 12^th^ hour. At the end of the 18^th^ hour, 4 and 23 patients in the granisetron and metoclopramide groups, respectively, complained about nausea, or at rates of 0.1 and 0.575 episodes/treatment (p = 0.0001).
- Emesis: In 15 chemotherapy treatments in the metoclopramide group, patients presented with vomiting in the 12^th^ hour (0.342 episodes/treatment), whereas 21 patients presented with this symptom in the 18^th^ hour (0.525 episodes/treat- ment) (p < 0.02). In the granisetron group, two patients presented with vomiting (0.15 episodes/treatment) in both the 12^th^ and 18^th^ hours, with no difference between these two groups. A statistically significant difference was observed in favor of the granisetron group, in both the 12^th^ and 18^th^ hours (p = 0.0004 and p = 0.0001, respectively).

The mean total scores from nausea, dry heaving and emesis were as follows: patients in the metoclopramide group had 11.525 episodes/treatment, whereas those in the granisetron group had six times fewer episodes than did the other group. The scores for the period between the 8^th^ and 18^th^ hours was seven times higher in the metoclopramide group (4.3 episodes/treatment) than in the granisetron group (0.6 episode/treatment), thus clearly showing the difference between these two drugs during the period when the patients were not in the hospital (Table 1).^1^

**Table 1:** Assessment of the efficacy of the antiemetics metoclopramide and granisetron according to different observation intervals following the beginning of chemotherapy treatment for osteosarcoma in children, and mean partial scores (numbers of episodes/patient of nausea, dry heaving and vomiting during a given period of time) in the MANE scale^18^

Interval (hours)	Total score	
	Metoclopramide	Granisetron
0 – 2	0.94	0.24
2 – 4	1.46	0.29
4 – 6	2.16	0.55
6 – 8	2.64	0.31
8 -12	1.92	0.28
12 -18	2.40	0.20
24 (total score)	11.52	1.87

Complete response, defined as absence of nausea, dry heaving and vomiting over the 24-hour period following the beginning of chemotherapy treatment, was observed in 62.5% and 10% of patients in the granisetron and metoclopramide groups, respectively. Table 2 shows the overall antiemetic efficacy according to response criteria.

**Table 2:** Overall antiemetic efficacy of metoclopramide and granisetron in children receiving chemotherapy for osteosarcoma according to response criteria (modified MANE^18^ scale)

Response	Points	Drug	
		Metoclopramide	Granisetron
Complete	0	(4/40) 10.0%	(25/40) 62.5%*
Partial	0 to 10	(14/40) 35.0%	(13/40) 32.5%
Minimum	11 to 20	(17/40) 42.5%	(2/40) 5.0%
Absence	More than 20	(5/40) 12.5%	(0/40) 0%
**Total**		**(40/40) 100%**	**(40/40) 100%**

* *p < 0.0001.*

Among the 26 patients included in the study, 16 could be compared to themselves (could be their "own controls") regarding antiemetic efficacy, because they received the same chemotherapy combination with two different antiemetic regimens. One patient, for instance, had a minimal response to metoclopramide but complete response to granisetron. On the other hand, another child had partial response to each of the two antiemetic regimens, and a third had complete response to both of them. However, the mean final scores for these 16 patients were 11.4 and 2.3 vomiting episodes/patient/24 hours when they received metoclopramide and granisetron, respectively (p = 0.02).

Twenty-four hours after infusion, 42.5% and 22.5% of the children administered the antiemetic regimens of metoclopramide plus dimenhydrinate and granisetron, respectively, refused to eat. Moreover, there were no reports of somnolence during the 24-hour assessment of the children in the granisetron group, whereas in the metoclopramide group 7.5% of the children presented somnolence, which in two cases consisted of torpor. Despite these findings, there was no statistically significant difference in relation to side effects between the two antiemetic regimens.

## DISCUSSION

We observed that the children and adolescents who received chemotherapy treatment according to the protocol for osteosarcoma adopted by our institution started to present with vomiting at a mean of two hours after beginning the infusion. The maximum emetic effect was reached between the 6^th^ and 8^th^ hours after starting their treatment. Despite receiving the antiemetic metoclopramide, these patients still presented with nausea in the 18^th^ hour after starting chemotherapy. Our results show that, in 62.5% of the patients undergoing chemotherapy treatments with granisetron, there was complete response, i.e. absence of nausea, dry heaving and emesis, whereas in the group treated with metoclopramide, complete response was achieved in only 10%. The proportions of all the chemotherapy treatments that were administered to patients who had complete response (not more than one episode of vomiting) were 80% and 27.5% for the granisetron and metoclopramide plus dimenhydrinate groups, respectively (p < 0.001).

Another matter that should be discussed relates to the differences in emetogenic potential between the three chemotherapy associations used in this study. The combination of epirubicin and carboplatin induced most of the vomiting episodes. Surprisingly, however, it was with this association that the biggest difference in complete response between the two antiemetic regimens was found: 64% of the patients in the granisetron group (7/11) had a complete response, whereas in the metoclopramide plus dimenhydrinate group, no patients presented complete response.

It was already known that granisetron was safe for administration to children; however, its dose had not yet been established. Tsuchida et al.^13^ reported on granisetron efficacy at doses of 20 µg/kg/day and 40 µg/kg/day, which were compared to determine the optimal dose of this antiemetic for children with solid tumors receiving high-dose chemotherapy. Granisetron at 40 µg/kg/day was more effective and patients did not present with significant side effects. On the basis of the dose recommended for adults, we adapted it for children in accordance with the body surface area, which thus suggested 50 µg/kg/day in a single dose. This dose was used here and was found to be safe and efficient. Our finding differs from the report by Lemerle et al.^14^ in which 24 patients were studied and the maximum dose of granisetron administered was 40 µg/kg/day. Their study assessed progressive doses of granisetron. However, it was conducted on patients with distinct pediatric tumors that required different chemotherapy regimens, which interfered with assessing the results adequately.

Zucker et al.^15^ coordinated a European multicenter study with 88 pediatric patients who were randomized to either granisetron 20 µg/kg, once to three times a day, or chlorpromazine 0.3 to 0.5 mg/kg plus dexamethasone 2 mg/kg per 8 hours. The chemotherapy treatment administered to these patients was iphosphamide 3 g/m^2^/day. Complete response was obtained for 50% of the patients that received granisetron, versus 19.5% of those treated with chlorpromazine plus dexamethasone. In the latter group, side effects such as somnolence, aplastic anemia and extrapyramidal reactions were observed. This study by Zucker et al.^15^ is important for having standardized the chemotherapy regimen, in addition to having gathered together a good number of cases. The mean number of emesis episodes in the granisetron group at a dose of 20 µg/kg (one to three doses/ day) was three times higher than the mean obtained in our study. The mean number of emesis episodes in their chlorpromazine plus dexamethasone group was 3.5 times higher than the mean found in the metoclopramide plus dimenhydrinate group in our study, which also showed lower incidence of side effects. Moreover, although we used a lower dose of iphosphamide (2,500 mg/m^2^/day), it was always in associations involving two chemotherapy drugs, which may have contributed towards increasing the mean number of vomiting episodes.

Regarding the number of patients, the antiemetic drugs utilized and the results obtained, our study can be compared to the one by Miyajima et al.^8^ 1994. These authors studied 22 patients with three chemotherapy regimens: a) ara-c, 3 g/m^2^; b) cisplatin, 50 mg/m^2^ ml/m^2^; c) iphosphamide 3 g/m^2^ plus actinomycin-D 900 µg/m^2^. These patients also received 40 µg/kg of granisetron or 2 mg/kg of metoclopramide. A complete response was found for 59.1% of the patients that received granisetron versus 0% of those that received metoclopramide.

Hirota et al.^16^ compared pediatric patients receiving granisetron versus granisetron plus methylprednisolone. In the latter group, more patients presented a complete response.

In another study by Jacobson et al.,^17^ 30 children received a dose of 20 µg/kg of granisetron. Sixty-six chemotherapy treatments were administered. In 35 (53%) of them, it was necessary to add one or two more doses of granisetron because the children still presented with nausea and vomiting. Their study suggested that it might be necessary to use additional doses of granisetron if the initial dose was low. In addition, it was conducted with 30 patients with 12 different diagnoses, who received one out of 11 distinct chemotherapy treatments with variable doses. In our study, we compared 26 patients with osteosarcoma who received the same therapeutic protocol in 80 chemotherapy treatments.

Another very important feature of our study concerns the parents' adherence to emesis assessments between the 8^th^ and 24^th^ hours. Interestingly, patients who did not present with emesis at the hospital also did not do so at home, and there were no exceptions. Those who presented with nausea and emesis at the hospital also did so at home, according to their parents.

## CONCLUSION

Administration of granisetron (50 µg/kg/ day) over a five-minute period proved to be six times more effective than an association of high doses of metoclopramide and dimenhydrinate (infused over an eight-hour period), for controlling chemotherapy-induced nausea and vomiting. It was found that 62.5% of the patients who received granisetron presented a complete response, with no significant side effects. Granisetron may be a good option for controlling nausea and vomiting in children receiving chemotherapy. The modified MANE scale was shown to be sensitive and easy to apply for assessing these symptoms in pediatric patients, even when treated as outpatients.
